# Homocysteine and Parkinson's disease

**DOI:** 10.1111/cns.14420

**Published:** 2023-08-29

**Authors:** Lingyan Zhou

**Affiliations:** ^1^ Department of Neurology Shandong Provincial Hospital Affiliated to Shandong First Medical University Jinan Shandong China

**Keywords:** homocysteine, hyperohomocysteinemia, Parkinson's disease

## Abstract

Homocysteine (Hcy) is an important metabolite in methionine metabolism. When the metabolic pathway of homocysteine is abnormal, it will accumulate in the body and eventually lead to hyperhomocysteinemia. In recent years, many studies have found that hyperhomocysteinemia is related to the occurrence and development of Parkinson's disease. This study reviews the roles of homocysteine in the pathogenesis of Parkinson's disease and illustrates the harmful effects of hyperhomocysteinemia on Parkinson's disease.

## Graphical abstract

Recent studies have found that hyperhomocysteinemia is related to the occurrence and progression of Parkinson's disease. Hcy may speed up the onset of Parkinson's disease through several mechanisms including inflammation, oxidative stress, and neuronal apoptosis.

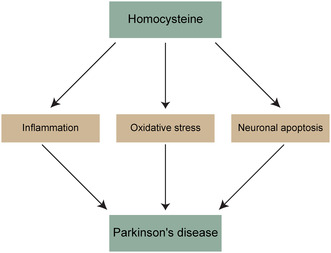

## 1 INTRODUCTION

Parkinson's disease (PD) is a common neurodegenerative disease for which there is no treatment modifying the course of the disorder and no reliable biomarkers for early diagnosis.^1^ It not only afflicts patients physically and mentally but also brings heavy economic and emotional burdens to their families and even society.^2^ In addition, with the extension of human life expectancy and the aging of the population, the number of people suffering from PD is increasing each year. According to a report in 2018, the number of PD patients in the world increased from 2.5 million in 1990 to 6.1 million in 2016. In just 26 years, the number of PD patients around the world has more than doubled.^3^ A relatively conservative prediction model shows that it is expected that there will be 12 million PD patients in the world by 2050. The author of this prediction model also points out that the quantity of PD patients will have a greater growth in the future if the aging of the population and the factors promoting PD continue to exist or worsen.^4^, ^5^ Thus, early detection and timely intervention of PD appear to be particularly important.

Homocysteine (Hcy) is an important intermediate product in methionine (Met) metabolism.^6^ Although Hcy does not directly participate in protein biosynthesis, it plays a role in maintaining Met and methylation levels in the body.^7^ When the level of Hcy increases abnormally, it leads to hyperhomocysteinemia (HHcy). HHcy has been indicated as a risk factor for a variety of disorders, such as cardiovascular diseases,^8^, ^9^, ^10^ neural tube defects,^11^, ^12^ atrial fibrillation,^13^, ^14^ Alzheimer's disease,^15^, ^16^ cancer,^17^, ^18^ multiple sclerosis,^19^, ^20^, ^21^ migraine,^22^, ^23^ and other diseases. Additionally, recent evidence showed that HHcy contributed to the development of PD.^24^, ^25^, ^26^ In this article, we discuss the relationship between Hcy and the pathogenesis of PD from three aspects: the structure of Hcy, the metabolism of Hcy, and the mechanism of HHcy in PD. This review aims to explore the roles of HHcy in inducing the occurrence and development of PD, laying a foundation for strategies to control the level of Hcy in the human body, which may contribute to the prevention and treatment of PD.

## 2 ORIGINS OF HCY

Hcy was first discovered in 1932 by Vincent du Vigneaud.^27^ It is a non‐essential α‐amino acid and an intermediate metabolite of the essential amino acid Met converted to cysteine. Hcy is not directly involved in protein synthesis and can only be synthesized through the metabolic pathway of Met in the body, and there is little Hcy in food.^28^ Both Hcy and its homologous cysteine contain a thiol group. The structural difference between them lies in the existence of an additional methylene bridge (‐CH2‐) in Hcy.^29^ Hcy is derived from Met and is synthesized by cleavage of Met terminal methyl group.^30^ Hcy can also be converted to Met or cysteine with the help of certain vitamins.

There are various types of Hcy in plasma. Most of it binds to plasma proteins. Some of it makes up Hcy dimers or dimers with other thiols. Very little of Hcy exists in the form of free thiol. The total Hcy in the plasm is composed of all the above‐mentioned forms of Hcy. These three different forms of Hcy account for about 70%–80%, 20%–30%, and 1% of the total plasma Hcy, respectively.^31^, ^32^ The human body has an intact regulation mechanism of Hcy metabolic pathway, which maintains the dynamic balance of Hcy content so that the total plasma Hcy level is maintained within a normal physiological range. The total plasma Hcy level is in the range of 5 to 15 μmol/L in the fasting state. Generally, the plasma Hcy level increases with age. And the capacity of plasma Hcy in men is often greater than that in women of the same age.^33^ Changes in some genetic or environmental factors can lead to an increase in plasma Hcy levels. When fasting total Hcy level in the plasma exceeds 15 μmol/L, this abnormal condition is called HHcy.^34^ HHcy can be divided into mild, moderate, and severe, with corresponding Hcy levels of 15–30 μmol/L, 30–100 μmol/L, and >100 μmol/L.^35^ Interestingly, a limited number of studies have found that there is a certain link between Hcy concentration and the accumulation of iron. R. Daher et al. found that serum iron content was positively correlated with serum Hcy concentration in 79 females (*r* = 0.321, *p* = 0.004).^36^ Christos Lioupis et al. reported that intraplaque iron burden was significantly associated with increased plasma Hcy levels in diabetic patients undergoing carotid endarterectomy.^37^ Moreover, another study found that there was a positive correlation between the plasma Hcy level and the amount of iron in the aorta of patients undergoing coronary artery bypass surgery (*r* = 0.657, *p* = 0.015).^38^

## 3 METABOLISM OF HCY

Hcy, a sulfur‐containing amino acid, is a byproduct of intracellular methylation and is produced by the metabolism of Met.^39^ Met can be supplied by food and is found in relatively higher amounts in foods such as eggs, sesame seeds, nuts, and meat. After being absorbed by epithelial cells in the digestive tract, Met can be transported to the organs of the human body. Met is metabolized mainly via the two reactions. (1) Methionine‐tRNA synthetase (MARS) catalyzed the aminoacylation of tRNA^Met^ to provide activated Met for new protein synthesis. (2) Methionine S‐adenosyltransferase (MAT) catalyzed Met to form S‐adenosylmethionine (SAM),^40^ After demethylation, SAM can be converted into S‐adenosylhomocysteine (SAH),^41^ which provides activated methyl groups for the methylation reaction in organisms and plays a crucial role in the synthesis of DNA, creatine, and phosphonate. SAH can then hydrolyze to form Hcy, the only known source of Hcy in the body.

**FIGURE 1:**
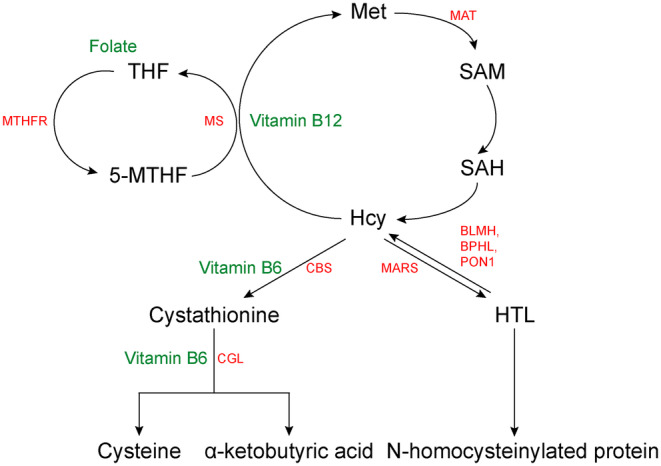
The metabolic pathway of methionine and homocysteine. 5‐MTHF, 5‐methyl tetrahydrofolate; BLMH, bleomycin hydrolase; BPHL, mitochondrial bisphenol hydrolase‐like; CBS, Cystathionine β‐synthase; CGL, cystathionine γ‐lyase; Hcy, homocysteine; HTL, homocysteine thiolactone; MARS, Methionine‐tRNA synthetase; MAT, Methionine S‐adenosyltransferase (MAT); Met, methionine; MS, Methionine synthase; MTHFR, 5, 10‐methylene tetrahydrofolate reductase; PON1, paraoxonase‐1; SAH, S‐adenosylhomocysteine; SAM, S‐adenosylmethionine; THF, tetrahydrofolate.

As shown in Figure 1, there are three metabolic pathways of Hcy: (1) Methionine synthase (MS) catalyzed the remethylation of Hcy to convert it into Met; (2) Cystathionine β‐synthase (CBS) and cystathionine γ‐lyase (CGL) catalyzed the conversion of Hcy to cysteine and α‐ketobutyric acid through the transsulfuration pathway; (3) MARS catalyzed Hcy to homocysteine thiolactone (HTL).

### 3.1 Remethylation pathway

When the level of Met in the body is low, Hcy metabolism mainly syntheses Met through remethylation to supply the deficient Met in the body. In this process, Hcy uses vitamin B12 (cobalamin) as a cofactor and 5‐methyl tetrahydrofolate (5‐MTHF) as the methyl donor produced by catalytic reactions mediated by 5, 10‐methylene tetrahydrofolate reductase (MTHFR).^42^ MS catalyzes the transfer of a methyl group from MTHF to Hcy, while converting MTHF to tetrahydrofolate (THF) and Hcy to Met. This process is also known as the Met cycle^43^ and can occur in any organ in the body.

### 3.2 Transsulfuration pathway

When Met is superfluous or when cysteine is needed, Hcy can be converted to cystathionine through the sulfur‐transfer pathway with vitamin B6 (pyridoxine) as the cofactor under the catalysis of CBS. And then cystathionine can be converted to cysteine and α‐ketobutyric acid under the catalysis of CGL. The excess cysteine can also be oxidized to taurine or treated with sulfation and excreted from the body.^27^, ^44^, ^45^ The sulfur‐transfer pathway of Hcy mainly occurs in the human liver,^46^ kidney,^47^ and brain.^48^ This is an effective mechanism to relieve organisms from the excessive amount of toxic sulfur‐containing amino acids. Most of the abnormal increase of Hcy is eliminated through this pathway.

### 3.3 Formation of HTL

The transformation process of Hcy to HTL catalyzed by MARS requires the participation of ATP,^49^ and this catalytic reaction can occur in various organs in the human body. When MTHFR,^50^ MS,^51^ or CBS^52^ defects caused by genetic factors damage the remethylation and sulfur‐transfer pathway of Hcy, or the Met‐rich diet increases the Hcy content,^53^ HTL generated by Hcy conversion will increase significantly.

HTL is metabolized in two ways. On the one hand, HTL is hydrolyzed and reconverted to Hcy. However, HTL can covalently modify the lysines of numerous proteins and thus cause changes in their activity. The hydrolytic pathway of HTL is catalyzed by homocysteine thiolactonase, such as bleomycin hydrolase (BLMH),^54^ mitochondrial bisphenol hydrolase‐like (BPHL),^55^ and paraoxonase‐1 (PON1).^56^ In the covalent‐modification pathway, HTL, as a highly reactive compound, reacts with lysine residues in the protein, resulting in N‐homocysteinylation of the protein, thus altering its structure and function.^57^, ^58^

## 4 MECHANISM OF HHCY IN PD

In recent years, many epidemiological investigations and clinical studies have revealed that Hcy levels of PD patients are significantly higher than those of healthy controls. And HHcy might be a high‐risk factor for PD.^59^, ^60^ Compared with PD patients without HHcy, PD patients with HHcy are more prone to depression and cognitive dysfunction.^61^, ^62^, ^63^ In a PD rat model induced by 6‐hydroxydopamine (6‐OHDA), rats with higher serum Hcy contents had more dopaminergic neuronal degeneration and more severe behavioral damage.^64^ These limited studies all indicate that there is a biological relationship between HHcy and PD, but the specific molecular mechanism is still confused and debated.

### 4.1 HHcy promotes inflammation in PD

The activation of glial cells and neuroinflammatory response play an important role in the pathogenesis of PD. Obvious inflammatory responses exist in the brain tissues of PD patients.^65^ One study found that Hcy could up‐regulate the expression of CD11b, which is a marker of microglia activation.^66^ Hcy can also induce the activation of nuclear factor kappa‐B (NF‐κB) in mouse brains, increase the release of IL‐1β, TNF‐α, and other inflammatory factors, and enhance the inflammatory response in brain tissue.^67^ Hcy can promote the proliferation and invasion of BV2 cells, and also promote the production of pro‐inflammatory cytokines in BV2 cells.^68^ Subsequent studies have found that Hcy can activate not only microglia but also astrocytes, thus enhancing neuroinflammatory response.^69^ Erica M. Weekman and colleagues separately cultured astrocytes and microglia in vitro and treated the cells with Hcy. Then quantitative reverse transcription polymerase chain reaction (qRT‐PCR) was used to detect gene expression. And it was found that the expression of genes associated with inflammation in astrocytes and microglia was significantly changed after Hcy treatment.^70^ In addition, other studies found that the immune reactivity of the astrocyte marker GFAP and the microglia marker IBA1 in the brain tissues of HHcy patients was significantly enhanced compared with those of healthy controls.^71^

### 4.2 HHcy induces oxidative stress in PD

Nivedita Bhattacharjee et al. injected Hcy into the brain of rats, and they discovered that the rats receiving Hcy injection had a strong oxidative stress response in the substantia nigra and striatum.^72^ After 30 days of subcutaneous injection of Hcy, the level of reactive oxygen species (ROS) and lipid peroxidation in the brain tissues of rats increased significantly.^73^, ^74^ After continuous 60 days of intraperitoneal injection of Hcy in mice, significant oxidative stress responses were also produced in the brain tissues of mice.^75^ Nadia Ferlazzo et al. found that the production of intracellular ROS of Neuro2a cells increased significantly, and the viability of Neuro2a cells decreased by about 40% after treatment with Hcy.^76^ It has been reported that the self‐oxidation process of Hcy may be the main mechanism of Hcy inducing ROS generation in brain tissue, but direct evidence of this mechanism is lacking at present.^77^ Other researchers have reported that Hcy‐induced oxidative stress response may be related to its induced changes in the antioxidant defense system. The key enzymes that compose the antioxidant defense system in the human body include superoxide dismutase (SOD), catalase, and glutathione peroxidase (GPx). Azadeh Aminzadeh et al. found that the total antioxidant capacity of H9C2 cells was significantly reduced after Hcy treatment in H9C2 cells.^78^ Aline Longoni et al. showed that Hcy treatment could reduce the activity of SOD and catalase in cells, but increase the activity of GPx.^79^ SOD, catalase, and GPx activities in the amygdala and prefrontal cortex were significantly enhanced in the rats with HHcy.^80^ However, it was also found that SOD activity in the cortex and hippocampus of rats treated with Hcy for a long time did not change significantly.^74^ Until now, the role of HHcy in antioxidant defense systems has been controversial. Some studies speculated that these different findings might be concerned with the different mechanisms of Hcy regulating the Keap1‐Nrf2 pathway under different conditions, thus leading to the activation or inhibition of antioxidant enzymes.^81^, ^82^

### 4.3 HHcy induces neuronal apoptosis in PD

Studies have found that Hcy can affect the growth and cell viability of human glioblastoma cells and promote cell apoptosis.^83^ Intraventricular injection of Hcy can lead to the death of dopaminergic neurons in rats.^84^, ^85^, ^86^ Hcy can exacerbate 1‐methyl‐4‐phenyl‐1,2,3,6‐tetrahydropyridine (MPTP)‐induced dopamine depletion, neuronal degeneration, and motor dysfunction.^87^ HHcy may mediate neuronal apoptosis in two main ways: (1) Promoting energy consumption; (2) Promoting DNA damage. After the injection of Hcy into rats, Hcy greatly reduces the uptake of glucose and the production of tricarboxylic acid cycle products by inhibiting the activities of succinate dehydrogenase and cytochrome C oxidase, resulting in brain energy metabolism disorders.^88^ Under physiological conditions, Hcy is converted to Met through the remethylation pathway, which depends upon folate and vitamin B12.^89^ Met plays a vital role in the metabolism of one carbon unit. It is involved in many biosynthetic processes and plays a crucial role in DNA synthesis, repair, and methylation.^6^ A deficiency of folic acid/vitamin B12 or excessive accumulation of Hcy can hinder the Met cycle, resulting in reduced methylation of DNA, which leads to the breaking of DNA strands. It has been reported that the mechanism of DNA damage in neurons may be related to the interruption of DNA methylation.^90^ HHcy can reduce the level of DNA methylation, thus interfering with gene transcription and DNA replication and damaging DNA repair, thus leading to cell apoptosis.^91^, ^92^ In addition, Hcy may also induce oxidative stress reactions, producing active nitrogen species and ROS, further damaging nerve cells and leading to apoptosis of nerve cells.

In addition, HHcy can induce neuronal apoptosis by inhibiting mitochondrial activity. One study found that Hcy‐mediated oxidative stress inhibited the activity of mitochondrial complex I, resulting in the dysfunction of mitochondrial respiratory chain function, which led to the death of dopaminergic neurons.^72^ Hcy can also enhance the activity of cysteinyl aspartate specific proteinase (caspase) and reduce the transmembrane potential of mitochondria, resulting in Ca^2+^ influx, calcium overload, and eventually apoptosis of nerve cells.^93^ Production of ROS and decreased activity of mitochondrial complex I might be an important mechanism of dopaminergic neuron apoptosis in PD.^94^

## 5 CONCLUSION

Hcy is a sulfur‐containing non‐essential amino acid. When the balance of Hcy metabolism is broken, Hcy accumulates in the human body, which leads to the occurrence of HHcy. HHcy is linked with the occurrence and progression of PD. This review briefly discussed the structure of Hcy, the metabolism of Hcy, and the mechanism of HHcy in PD. There are many disputes about the relationship between HHcy and PD which remains to be investigated. This review aims to discuss the mechanisms of harmful effect of HHcy on PD. Further studies are needed to provide more convincing evidence demonstrating the pathogenic role of HHcy in the progression of PD in animal and clinical studies. Besides mentioned facts, it remains to be examined whether Hcy is a causative agent or marker of damage.

## FUNDING INFORMATION

The authors received no financial support for this article.

## CONFLICT OF INTEREST STATEMENT

The authors report no declarations of interest.

## Data Availability

Data sharing not applicable to this article as no datasets were generated or analysed during the current study.
